# Cardiac Resynchronization Therapy in a Patient with Persistent Left Superior Vena Cava Draining into the Coronary Sinus and Absent Innominate Vein: A Case Report and Review of Literature

**DOI:** 10.1016/s0972-6292(16)30799-9

**Published:** 2014-10-06

**Authors:** Girish M Nair, Seeger Shen, Pablo B Nery, Calum J Redpath, David H Birnie

**Affiliations:** Arrhythmia Service, Division of Cardiology, University of Ottawa Heart Institute, 40 Ruskin Ave, Ottawa, Canada - K1Y 4W7

**Keywords:** Cardiac Resynchronization Therapy, Persistent Left Superior Vena Cava, Coronary Sinus, Absent Innominate Vein

## Abstract

**Introduction:**

Persistent left superior vena cava (PLSVC) is a rare congenital anomaly of the superior venous system that may be discovered at the time of cardiac implantable electronic device (CIED) implantation.

**Methods and Results:**

We present a subject who needed cardiac resynchronization therapy (CRT)-CIED implantation and was discovered to have PLSVC with absent innominate vein during the implant procedure. We were able to successfully implant a CRT-CIED using a right-sided approach via the right superior vena cava (SVC). We present a description of our implant technique and a brief review of the different aspects of CIED implantation in subjects with variants of PLSVC.

**Conclusion:**

Superior venous anomalies such as PLSVC can make CIED implantation technically challenging. However, with increasing operator experience, cardiac imaging and appropriate tools successful CIED implantation is possible in almost all cases.

## Introduction

Persistent left superior vena cava (PLSVC) is a congenital venous developmental abnormality of the sinus venosus with an incidence of 0.47% in patients undergoing cardiac implantable electronic devices. The two variants include a double superior vena cava (right and left SVC, with or without an innominate vein connecting the two) or a single left sided SVC (without a right SVC) [1]. Implantation of cardiac implantable electronic devices (CIED), especially cardiac resynchronization therapy (CRT), may be challenging in the presence of PLSVC [2]. We present our approach to CRT-CIED implantation in a subject with no previous documentation of systemic superior venous anomalies or congenital heart disease.

## Case Report

**Figure 1:**
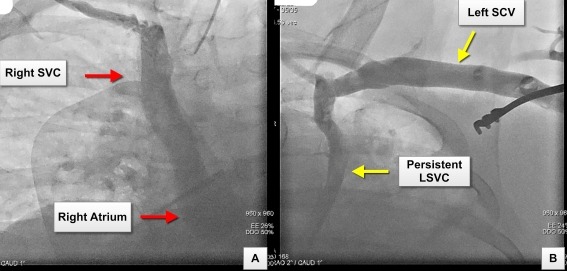
Panel A: Venogram showing the course of the right superior vena cava (SVC) draining into the right atrium Panel B: Venogram showing the left subclavian vein (SCV) draining into a persistent left superior vena cava (PLSVC). The venogram also demonstrates the absence of the innominate vein

A 59-years old gentleman with non-ischemic dilated cardiomyopathy, severe LV systolic dysfunction (left ventricular ejection fraction = 25%), LBBB (QRS width 180 ms), NYHA class III symptoms (on optimal medical management) and morbid obesity (body weight 160 kg) was referred to our service for CRT-CIED insertion. The procedure was performed under general anesthesia and full therapeutic anticoagulation as the subject had a history of paroxysmal atrial fibrillation with CHADS2 score of 3. A left superior venogram performed to guide left subclavian venous access revealed the presence of a PLSVC draining into the proximal segment of the coronary sinus (CS). The combined PLSVC and CS confluence drained into the right atrium. The venogram also revealed the absence of an innominate vein connecting the PLSVC to the right SVC. A venogram performed from the right side demonstrated the presence of a right SVC draining into the right atrium (Figure 1).

**Figure 2:**
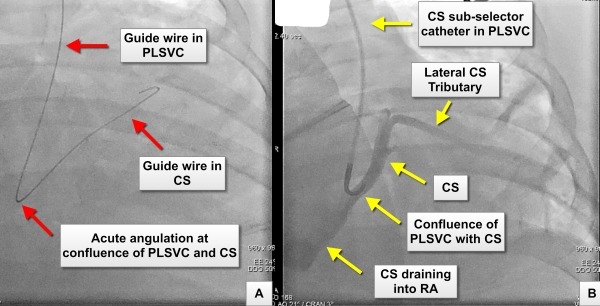
Panel A: Image showing the course of a J-tip 0.032" hydrophilic guide wire through the persistent left superior vena cava (PLSVC) into the coronary sinus (CS). The acute angulation of the coronary guide wire at the confluence of the PLSVC and CS is shown. This anatomical feature may make cannulation and delivery of a CS pacing lead technically challenging Panel B: Venography using a CS sub-selector catheter, positioned at the confluence of the PLSVC with the CS, demonstrate the anatomy of the CS and the presence of a lateral CS tributary suitable for pacing lead placement

The left subclavian vein was accessed using the Seldinger technique and a CS sub-selector catheter (Medtronic Attain Select soft-tipped guide catheterTM ) was introduced over a guide wire to cannulate the coronary sinus, distal to its confluence with the PLSVC. The selective coronary sinus venogram demonstrated a suitable lateral tributary for coronary sinus pacing lead insertion (Figure 2). However, we felt that a left sided approach to implant three pacing leads, especially in the presence of acute angulation between the PLSVC and the CS, would be technically challenging and associated with a high risk of lead dislodgement. In addition the presence of a right-sided SVC draining into the right atrium prompted us to attempt device implantation using a right-sided approach.

**Figure 3:**
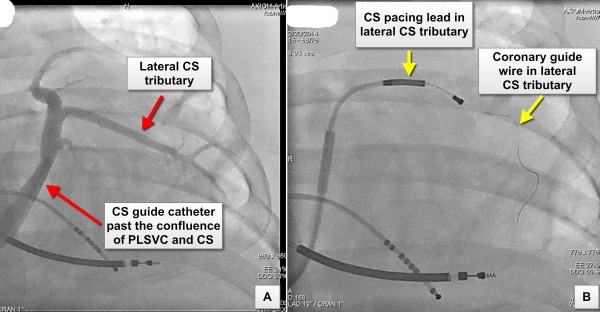
Panel A: Coronary sinus (CS) venogram performed after cannulation using a guide catheter introduced via the right superior vena cava (SVC). The tip of the guide catheter has been positioned distal to the confluence of the persistent left superior vena cava (PLSVC) and the CS. In view of the relative narrow caliber of the CS an occlusive venogram was not performed Panel B: Image showing a bipolar 6F endocardial pacing lead over a 0.014" coronary guide wire being introduced into the lateral CS tributary

**Figure 4:**
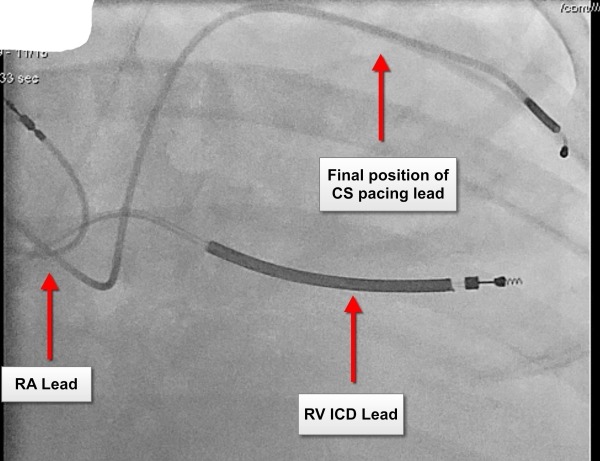
Final position of pacing leads in the heart. RA- Right atrium; RV- Right Ventricle; CS- Coronary sinus

We were able to successfully implant a CRT-CIED system using a right-sided approach. A straight coronary sinus cannulation catheter (Medtronic 7F catheterTM) was introduced over a deflectable decapolar electrophysiological diagnostic catheter into the CS, distal to its confluence with the PLSVC. A venogram demonstrated the presence of a lateral CS tributary of adequate caliber for CS pacing lead placement. An endocardial bipolar pace-sense CS lead (Medtronic 6F 4194 leadTM) was introduced over a 0.014" coronary guide wire (WHISPER extra-support; Abbott VascularTM) into this tributary (Figure 3 and 4). The subject withstood the procedure well without any acute complications and was discharged home.

## Discussion

Our case highlights a rare congenital venous anomaly that could potentially make CIED implantation technically challenging. The important factors increasing the complexity of lead placement from the left side include the tendency for the RV lead to be deflected away from the tricuspid annulus, torrential CS blood flow, acute angulation at the point where the PLSVC joins the CS and other anomalies making lead placement extremely difficult [3-5]. In our case PLSVC, without an innominate vein connecting it to the right SVC, made it difficult for us to implant a CRT-CIED using a left sided approach. We decided to attempt CIED implantation from the left side only if a right-sided approach failed. The presence of a right subclavian vein draining into the right SVC allowed us the opportunity to successfully implant the device using a right-sided approach. In addition favorable CS anatomy with the presence of a discrete normal sized CS body distal to its confluence with the PLSVC and an adequate sized lateral CS tributary facilitated left ventricular (LV) pacing lead insertion using the endovascular approach. Another option would have been to tunnel all three pacing leads from the right side to the left side in order to avoid placing the CRT-CIED on the right side. However, we decided against this option, in this morbidly obese subject, considering the lack of data regarding longevity of tunneled leads, the technical challenges associated with tunneling three leads across the midline and possibly extracting them at a future date, in the event of device infection or lead malfunction. Other operators have been unsuccessful in implanting a CS pacing lead from the right side, in a subject with PLSVC variant identical to our case, due to torrential CS blood flow and difficult anatomy. They had to insert the CS pacing lead from the PLSVC and tunnel the lead to the right side [5].

The presence of PLSVC can be inferred from the presence of dilated CS on echocardiography. If the presence of PLSVC is suspected prior to CIED insertion cardiac imaging (CT/MRI/3-D echo) can be used to understand superior venous anatomy. This will assist the operator in selecting the most appropriate approach and the necessary tools (stylets, leads, cannulation catheters and guidewires) for CIED implantation, possibly increasing the chance of success. In view of the very low incidence of PLSVC, in subjects without congenital heart disease, routine cardiac imaging to detect superior venous anomalies in patients may not be indicated. Given the higher incidence (10-15%) superior venous anomalies in subjects with congenital heart disease a case can be made for cardiac imaging prior to CIED insertion, especially in subjects needing CRT [1,2]. Torrential CS blood flow may necessitate pressure injection of radiocontrast and large-sized balloon catheters to perform venography to delineate cardiac chambers, CS-PLSVC confluence and CS tributaries [2,3].

The variant of PLSVC without a right SVC or innominate vein connecting the PLSVC to the right atrium makes LV pacing lead insertion via the CS very difficult. In such subjects experienced operators have used a variety of special stylets, catheters and pacing leads to successfully implant CRT-CIED using an endovascular left-sided approach [2-4]. Other operators have adopted a hybrid approach implanting atrial and ventricular pacing leads via an endovascular approach and LV epicardial pacing lead using an open or minimally invasive surgical approach [1].

The variant of PLSVC with right-sided SVC, with or without an innominate vein connecting the right and left SVC, makes it more likely that a CRT-CIED can be inserted exclusively using an endovascular approach. In this situation operators have used right or left sided approaches to insert all three pacing leads (RA, RV and CS). Other operators have used a hybrid approach inserting the RA and RV pacing leads from the right side and the CS pacing lead from the left side using the PLSVC. The CS pacing lead was then tunneled through the subcutaneous plane to the right side [5]. Very rarely the PLSVC may be associated with an unroofed CS and an atrial septal defect. In this situation life-long systemic oral anticoagulation is used to prevent systemic thromboembolism [1].

## Summary

Superior venous anomalies such as PLSVC can make CIED implantation technically challenging. However, with increasing operator experience, cardiac imaging and appropriate tools successful CIED implantation is possible in almost all cases.
